# Hospitalized Patients with Oral Cavity Cancer and Ulcerative Mucositis: Implications for Key Cost Drivers and Disparities

**DOI:** 10.3390/reports9030203

**Published:** 2026-06-26

**Authors:** Lauryn Rudin, Roberto Pili, Joel B. Epstein, Karrar Aljanahi, Diggory Cordova, Richa Rajesh, Kapil Meleveedu, Poolakkad S. Satheeshkumar

**Affiliations:** 1 Department of Medicine, University at Buffalo, Buffalo, NY 14203, USA; laurynru@buffalo.edu; 2 Division of Hematology and Oncology, Department of Medicine, University at Buffalo, Buffalo, NY 14203, USA; rpili@buffalo.edu; 3 City of Hope Comprehensive Cancer Center, Duarte CA and Samuel Oschin Comprehensive Cancer Institute, Cedars-Sinai Medical System, Los Angeles, CA 90048, USA; jepstein@coh.org; 4 Jacobs School of Medicine and Biomedical Sciences, Buffalo, NY 14203, USA; karraral@buffalo.edu (K.A.); diggoryc@buffalo.edu (D.C.); 5 College of Dental Medicine, Nova Southeastern University, Davie, FL 33328, USA; rr2144@mynsu.nova.edu; 6 UConn Health: Neag Comprehensive Cancer Center, University of Connecticut, 135 Dowling Way 4th Floor, Farmington, CT 06030, USA; kmeleveedu@uchc.edu

**Keywords:** cancers of the lip, oral cavity, and pharynx, chemotherapy-induced ulcerative mucositis, radiotherapy-induced ulcerative mucositis, burden of illness, length of stay, health disparities, National Inpatient Sample

## Abstract

Background: Cancer treatment-induced ulcerative mucositis (UM) is a debilitating toxicity in patients with cancers of the lip, oral cavity, and pharynx (CLOP). This study evaluated the association of chemotherapy-induced (CT-UM) and radiotherapy-induced ulcerative mucositis (RT-UM) with burden of illness (BOI), focusing on hospital length of stay (LOS) and total charges, and examined disparities in outcomes. Methods: This retrospective cohort study analyzed 2019 National Inpatient Sample (NIS) data. Adult patients (≥18 years) hospitalized with CLOP (ICD-10-CM C00–C14) undergoing inpatient surgery, chemotherapy, or radiotherapy were included. CT-UM (K12.31) and RT-UM (K12.33) were identified as secondary diagnoses. Survey-weighted generalized linear models (negative binomial for LOS; gamma for charges) adjusted for demographics, comorbidities (Elixhauser score), insurance, income, and Diagnosis-Related Groups (DRG; surgical vs. medical) were used. Results: Among 59,710 weighted CLOP hospitalizations, 820 had CT-UM and 1010 had RT-UM. Patients with UM were younger and had varying comorbidity burdens. Unadjusted analyses showed prolonged geometric mean LOS for CT-UM (5.66 vs. 3.81 days, *p* < 0.001) and RT-UM (4.95 vs. 3.81 days, *p* = 0.001), with lower total charges ($48,645 and $42,938 vs. $56,267). Multivariable analyses confirmed RT-UM was associated with increased LOS (adjusted coefficient 1.33, 95% CI 1.14–1.55) but lower charges (0.67, 95% CI 0.56–0.81). In patients >50 years, CT-UM showed stronger effects (LOS 1.80, 95% CI 1.49–2.15; charges 0.79, 95% CI 0.65–0.98). Significant disparities were observed: females, Black and Hispanic patients, and Medicaid beneficiaries experienced greater BOI (prolonged LOS and/or higher charges in subgroups). Associations persisted in DRG- and procedure-stratified sensitivity analyses, suggesting treatment interruptions as a key driver. Conclusions: Ulcerative mucositis in hospitalized CLOP patients is associated with prolonged LOS but lower charges, likely due to treatment modifications, and disproportionately affects vulnerable populations. These findings highlight the need for proactive oral care protocols, multidisciplinary integration, and equity-focused interventions to reduce the burden of this toxicity and improve cancer treatment outcomes.

## 1. Introduction and Clinical Significance

Cancer treatment-induced ulcerative mucositis (UM) is a major dose-limiting toxicity of chemotherapy (CT) and radiotherapy (RT) in patients with cancers of the lip, oral cavity, and pharynx (CLOP) [1,2,3,4]. It severely impairs quality of life, nutrition, and treatment tolerance, often causing unplanned interruptions, dose reductions, and increased healthcare utilization [1,5,6,7,8]. Prospective studies in head and neck squamous cell carcinoma (HNSCC) show that grade 3 toxicities and weight loss during RT or chemoradiotherapy are primary drivers of treatment breaks, with UM serving as a strong predictor in both RT and combined regimens [2,6,7,8]. Oral ulcerative mucositis must be differentiated from other causes of oral ulceration, including infectious stomatitis (e.g., herpes simplex virus and candidiasis), autoimmune conditions (oral lichen planus and pemphigus), traumatic or aphthous ulcers, and systemic manifestations such as graft-versus-host disease (GVHD) in transplant recipients [3,9,10]. Pre-existing conditions like poor dentition or periodontal disease can exacerbate or mimic these changes, highlighting the need for baseline dental assessment [3,9,10].

Mucositis-related treatment interruptions remain underexplored in many trials despite their impact [7,8]. Hospitalization rates for radiotherapy complications, including severe mucositis, rose 1.3-fold from 2005 to 2016 [11]. In real-world settings, UM drives prolonged length of stay (LOS), higher costs, and outcome disparities, especially in underserved groups [12,13,14]. Systemic effects include dehydration, malnutrition, sepsis, cachexia, and reduced survival, amplified in CLOP patients receiving multimodal therapy [1,3,12,13]. This short report examines the association of chemotherapy-induced (CT-UM) and radiotherapy-induced ulcerative mucositis (RT-UM) with burden of illness (BOI)—primarily LOS and total hospital charges—among hospitalized CLOP patients using 2019 National Inpatient Sample (NIS) data. We hypothesized that UM contributes to adverse outcomes, including treatment modifications and disparities by age, sex, race/ethnicity, insurance, and socioeconomic status.

Administrative datasets like NIS capture severe inpatient cases but may underestimate milder outpatient-managed mucositis. By applying specific ICD-10-CM codes (C00–C14 for CLOP; K12.31 for CT-UM; K12.33 for RT-UM) in treatment contexts, this analysis offers valuable insights into inpatient burden. Such evidence supports multidisciplinary preventive strategies, including proactive oral care, photobiomodulation, nutritional support, and risk-stratified interventions, to improve therapy tolerance, quality of life, and health equity [15,16,17,18,19]. Integrating oral medicine specialists into oncology and palliative teams is essential. Baseline optimization, validated monitoring (e.g., MASCC/ISOO scales), and culturally tailored care can mitigate toxicity and reduce systemic/economic burdens in vulnerable populations [12,13,14,15,17,19].

## 2. Methods

This retrospective cohort study analyzed data from the 2019 National Inpatient Sample (NIS), the largest publicly available all-payer inpatient database in the United States, maintained by the Agency for Healthcare Research and Quality (AHRQ) as part of the Healthcare Cost and Utilization Project (HCUP) [20]. The NIS is a 20% stratified sample of hospitalizations from community hospitals, designed to produce nationally representative estimates when appropriate discharge weights are applied [20].

Inclusion and Exclusion Criteria: Adult patients (≥18 years) hospitalized with cancers of the lip, oral cavity, and pharynx (CLOP; ICD-10-CM codes C00.0–C14.9) who underwent inpatient surgery, chemotherapy, or radiotherapy were included. Chemotherapy-induced ulcerative mucositis (CT-UM) was identified by the secondary diagnosis code K12.31 (Oral mucositis [ulcerative] due to antineoplastic therapy) and radiotherapy-induced ulcerative mucositis (RT-UM) by K12.33 (Oral mucositis [ulcerative] due to radiation). These codes were restricted to index admissions involving relevant treatment procedures (identified via ICD-10-PCS and CCS codes) to ensure context of treatment-related toxicity. Exclusion criteria included patients under 18 years of age, elective admissions lacking treatment codes, and cases where mucositis was coded as the primary diagnosis without associated antineoplastic therapy. Survey weights were applied throughout to generate national estimates [20].

Data Elements and Outcomes: The primary outcomes representing burden of illness (BOI) were hospital length of stay (LOS) in days and total hospital charges (in US dollars). Covariates included patient demographics (age, sex, race/ethnicity, insurance status, median household income quartile); clinical factors (weighted Elixhauser comorbidity index); hospital characteristics (bed size, teaching status, region, urban-rural location); and admission-related variables (Diagnosis-Related Group [DRG] category: surgical vs. medical; major head and neck procedures) [20]. Race/ethnicity was categorized as White, Black, Hispanic, Asian/Other based on NIS documentation.

Statistical Analysis: Univariate comparisons between groups (with vs. without CT-UM or RT-UM) were performed using Rao-Scott chi-square tests for categorical variables and *t*-tests for continuous variables, accounting for the complex survey design. Multivariable survey-weighted generalized linear models (GLMs) were employed: negative binomial regression for LOS (to handle over-dispersion) and gamma distribution with log link for total charges. Models were adjusted for the covariates listed above, with inclusion of interaction terms where relevant (e.g., age × UM status). Prespecified subgroup analysis was conducted for patients older than 50 years. Sensitivity analyses included DRG stratification, major procedure adjustment, and broader mucositis codes (K12.30, K12.32). All analyses were two-tailed, with statistical significance set at *p* < 0.05. Results are reported as adjusted coefficients with 95% confidence intervals (CIs).

This study was exempt from Institutional Review Board (IRB) review because it utilized publicly available, de-identified data consistent with HCUP’s limited dataset provisions under the HIPAA Privacy Rule [20]. No patient consent was required.

Limitations: The NIS captures only inpatient encounters, potentially underestimating milder outpatient-managed cases. Reliance on administrative coding may result in under-ascertainment of mucositis severity, and detailed clinical variables such as dental status, specific treatment regimens, or outpatient follow-up are unavailable.

## 3. Results

In 2019, a total of 59,710 weighted hospitalizations involving patients with cancers of the lip, oral cavity, and pharynx (CLOP; ICD-10-CM C00–C14) who underwent inpatient surgery, chemotherapy, or radiotherapy were identified in the National Inpatient Sample. Among these, 820 patients had chemotherapy-induced ulcerative mucositis (CT-UM; K12.31), and 1010 had radiotherapy-induced ulcerative mucositis (RT-UM; K12.33) documented as secondary diagnoses during the index admission.

Description of Baseline Characteristics (Table 1): Table 1 presents the weighted baseline characteristics of CLOP patients stratified by the presence of CT-UM or RT-UM. Patients with CT-UM were significantly younger than those without (mean age 61.84 ± 11.40 vs. 64.49 ± 12.24 years, *p* = 0.004) and exhibited higher comorbidity burden as measured by the weighted Elixhauser score (18.02 ± 8.66 vs. 16.22 ± 9.80, *p* = 0.009). Demographically, the CT-UM group had a lower proportion of females (23.8% vs. 29.6%, *p* = 0.12), higher representation of White patients (82.5% vs. 76.0%), and notably higher rates of private insurance (39.6% vs. 27.9%). They were also more likely to reside in higher income quartiles and less likely to be transferred out of the hospital (9.1% vs. 18.9%, *p* < 0.001).

Similar trends were observed in the RT-UM cohort, though age difference was not statistically significant (63.50 ± 11.87 vs. 64.47 ± 12.24 years, *p* = 0.24). RT-UM patients also showed higher White race proportion (83.6% vs. 75.9%) and private insurance coverage (31.2% vs. 28.0%). Comorbidity scores were comparable (16.95 ± 8.96 vs. 16.23 ± 9.80, *p* = 0.26). Both UM groups had lower rates of hospital transfer-out, potentially reflecting more complex in-hospital management needs. Patient location (urban-rural) and other hospital characteristics showed minor variations without consistent patterns across groups. These baseline differences underscore the importance of multivariable adjustment in subsequent analyses.

Unadjusted Analyses: Unadjusted outcomes revealed substantial burden associated with ulcerative mucositis. For CT-UM, geometric mean length of stay (LOS) was markedly prolonged by 5.66 days compared to 3.81 days in non-CT-UM patients (*p* < 0.001). Total hospital charges were lower ($48,645 vs. $56,267, *p* = 0.05). For RT-UM, LOS was 4.95 days vs. 3.81 days (*p* = 0.001), with even lower charges ($42,938 vs. $56,267, *p* < 0.001). These patterns suggest that while mucositis extends hospitalization duration—likely due to supportive care needs, nutritional support, pain management, and treatment interruptions—it is associated with reduced daily procedural intensity and overall charges, possibly from de-escalation of antineoplastic therapy.

**Figure 1:**
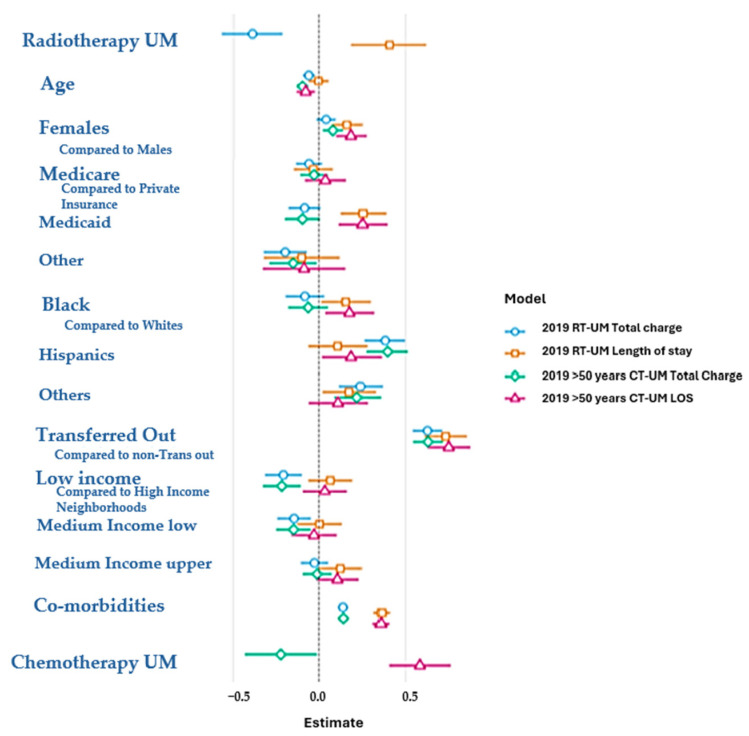
Generalized linear models estimating total charge variation among ulcerative mucositis (UM) patients.

Adjusted Multivariable Analyses (Figure 1): The figure represents survey-weighted generalized linear models (GLMs) with appropriate distributions (negative binomial for LOS; gamma with log link for charges) that were adjusted for key confounders, including age, sex, race/ethnicity, insurance status, median household income quartile, weighted Elixhauser comorbidity score, hospital teaching status, bed size, region, and Diagnosis-Related Group (DRG) category (surgical vs. medical). Sensitivity analyses further incorporated major head and neck procedure indicators (ICD-10-PCS) and interaction terms.

RT-UM remained independently associated with increased LOS (adjusted coefficient 1.33, 95% CI 1.14–1.55) but significantly lower total charges (coefficient 0.67, 95% CI 0.56–0.81). Overall, CT-UM was associated with longer LOS (coefficient 1.54, 95% CI 1.36–1.74). In the prespecified subgroup analysis of patients older than 50 years, CT-UM demonstrated amplified effects: LOS coefficient 1.80 (95% CI 1.49–2.15) and reduced charges (coefficient 0.79, 95% CI 0.65–0.98). These associations were robust in DRG-stratified and procedure-adjusted models, supporting the interpretation that mucositis-related treatment interruptions contribute independently to prolonged supportive care admissions with lower per diem costs.

Disparities in Burden of Illness: Important disparities emerged. Females had consistently longer LOS across both UM cohorts (adjusted coefficient 1.13, 95% CI 1.06–1.19). Racial/ethnic differences were pronounced: Hispanic patients experienced higher total charges in RT-UM (coefficient 1.48, 95% CI 1.30–1.64) and older CT-UM subgroups (coefficient 1.46, 95% CI 1.31–1.67), along with longer LOS in the latter (coefficient 1.21, 95% CI 1.02–1.43). Black patients showed prolonged LOS in RT-UM (coefficient 1.17, 95% CI 1.01–1.35) and older CT-UM cohorts (coefficient 1.19, 95% CI 1.04–1.38) compared to White patients. Medicaid beneficiaries had independently longer LOS (coefficient 1.19 in RT-UM; 1.29 in older CT-UM), highlighting systemic barriers such as post-acute care placement challenges.

All analyses were two-tailed, with statistical significance set at *p* < 0.05. Table 1 provides complete weighted frequencies, percentages, and *p*-values for all variables. These findings quantify the substantial inpatient burden of ulcerative mucositis in CLOP and emphasize equity considerations.

## 4. Discussion

This nationwide analysis of hospitalized patients with cancers of the lip, oral cavity, and pharynx (CLOP) demonstrates that ulcerative mucositis (UM) is associated with prolonged hospital length of stay (LOS) but also with lower total charges, likely reflecting clinically meaningful treatment interruptions or modifications due to toxicity. These findings align with prior evidence that UM is a dose-limiting toxicity leading to unplanned breaks in radiotherapy (RT) and chemotherapy (CT), which compromise disease control and increase supportive care needs [1,3,7,8,12,13,21]. The paradoxical combination of extended LOS with reduced charges is particularly noteworthy. While longer hospitalizations intuitively increase costs, our multivariable models—adjusted for Diagnosis-Related Groups (DRG; surgical vs. medical), major head and neck procedures, and other confounders—show that RT-UM and, in older patients, CT-UM are independently linked to this pattern. This suggests that mucositis often prompts de-escalation of high-intensity antineoplastic interventions (e.g., reduced RT fractions, delayed or omitted CT cycles) in favor of prolonged supportive care admissions focused on pain management, nutritional support, hydration, and infection control [12,13,22,23,24,25]. Such interruptions are well-documented in prospective head and neck cohorts, where grade 3+ mucositis correlates with higher rates of treatment breaks [3,7,8].

Local and Systemic Factors Influencing Mucositis: Both local and general factors significantly modulate mucositis severity and downstream outcomes. Local factors include poor dentition, periodontal disease, pre-existing oral infections, sinusitis, and chronic irritation from ill-fitting prostheses or xerogenic medications. These can exacerbate mucosal injury, impair healing, and increase susceptibility to secondary bacterial or fungal infections [4,15,17,19]. For instance, poor oral hygiene and dental foci have been associated with higher mucositis incidence and complications such as febrile neutropenia in cancer patients [22,23,26]. Systemic contributors encompass advanced age, comorbidities (e.g., diabetes, malnutrition), smoking, dehydration, low body mass index, and pharmacogenomic variations affecting drug metabolism or mucosal repair [1,4,21,26,27].

In our cohort, higher Elixhauser comorbidity scores in UM patients support the role of these multifactorial influences. Unfortunately, administrative datasets like the NIS lack granular data on baseline dental status or specific medication use, representing a key limitation [20]. Future studies integrating electronic health records with dental evaluations could better quantify these contributions. Clinically, proactive pre-treatment dental optimization and management of sinusitis or xerogenic drugs remain critical, consistent with MASCC/ISOO guidelines [15,17,19].

Disparities in Burden of Illness: Our analysis highlights pronounced disparities. Women, Black and Hispanic patients, and Medicaid beneficiaries experienced greater increases in LOS and, in some subgroups, charges [21]. These patterns likely stem from structural barriers—including limited access to outpatient supportive care, transportation challenges, lower health literacy, food insecurity, and implicit bias in pain/discharge planning—compounded by higher baseline comorbidity burdens in underserved populations [21,28,29,30,31,32]. Medicaid insurance, more prevalent among minority groups, is associated with post-acute care placement delays, prolonging acute hospitalizations [28,29,32].

Biologically, differences in mucosal repair capacity, pharmacogenomics, and comorbidity profiles (e.g., higher diabetes and cardiovascular disease prevalence) may amplify toxicity risk. These disparities extend broader inequities in head and neck cancer outcomes, where Black patients often face worse survival [30,31]. Our findings underscore the urgent need for culturally tailored interventions, such as community-based oral care navigation programs (e.g., through initiatives like NICE), to mitigate these gaps [21,31].

Clinical and Economic Implications: The inpatient focus of this study captures the most severe, resource-intensive UM cases. While overall charges appear lower due to treatment de-escalation, the true economic burden is substantial when considering downstream effects: increased infection risk, readmissions, and lost productivity [12,13,14,22,23,24,25,33]. Prior estimates place the incremental cost of severe mucositis at $5000–$30,000 per patient in RT settings and even higher in transplant populations. Preventing severe UM could yield significant savings and improve oncologic outcomes by minimizing treatment interruptions [12,15,16,17,18,25]. The strengths of this study include its use of a large, nationally representative dataset, specific ICD-10-CM coding for CT-UM versus RT-UM, robust adjustment for confounders including DRG, and explicit examination of disparities. Limitations include potential under-coding of milder cases, lack of outpatient data, absence of detailed treatment dosimetry or dental variables, and the cross-sectional nature of NIS records, which precludes causality assessment [20].

Future Directions and Recommendations: These results support the implementation of standardized proactive oral care protocols: baseline dental evaluation, weekly mucositis risk screening with validated tools (e.g., MASCC/ISOO scales), photobiomodulation therapy, high-potency rinses, cryotherapy, and early multidisciplinary involvement of oral medicine specialists, nutritionists, and palliative care teams [15,16,17,18,19]. Risk-stratified pathways for high-risk groups (older adults, racial/ethnic minorities, and Medicaid-insured) should incorporate pre-treatment optimization and nutritional rehabilitation [21,28,29,30,31,32]. Hospitals should adopt quality metrics tracking mucositis-related LOS and treatment interruptions. Future research should link NIS with outpatient claims, incorporate real-world dental and salivary biomarker data, and evaluate AI-driven predictive models (e.g., nomograms) for personalized prevention [34,35]. Longitudinal studies examining long-term survival impacts of UM-driven interruptions are also warranted [7,8,9,10,27].

## 5. Conclusions

Ulcerative mucositis in CLOP patients drives prolonged hospitalizations and reveals important disparities. By addressing modifiable local and systemic factors and implementing equitable supportive care strategies, clinicians and health systems can reduce the burden of this debilitating toxicity, improve treatment tolerance, and advance health equity in oncology.

## Figures and Tables

**Table 1:** Baseline characteristics of patients with CLOP in 2019 with and without ulcerative mucositis.

	Patients Without Chemo-Ulcerative Mucositis * (Weighted)	Patients with Chemo-Ulcerative Mucositis * (Weighted)	Patients Without RT-Ulcerative Mucositis * (Weighted)	Patients with RT-Ulcerative Mucositis * (Weighted)
n	58,890	820	58,700	1010
Age (mean (SD *))	64.49 (12.24)	61.84 (11.40) *p* = 0.004	64.47 (12.24)	63.50 (11.87) *p* = 0.24
Sex (%)				
Female	17,435.0 (29.6)	195.0 (23.8) *p* = 0.12	17,330 (29.5)	300 (29.7) *p* = 0.96
Race (%)				
White	43,835.0 (76.0)	660.0 (82.5)	43,680 (75.9)	815 (83.6)
Black	6125.0 (10.6)	55.0 (6.9)	6115 (10.6)	65 (6.7)
Hispanic	3845.0 (6.7)	35.0 (4.4)	3820 (6.6)	60 (6.2)
Asian and Others	3905.0 (6.8)	50.0 (6.3) *p* = 0.22	3920 (6.8)	35 (3.6) *p* = 0.66
Median household income (based on current year)				
0–25th percentile	16,665.0 (28.8)	145.0 (17.9)	16,555 (28.7)	255 (25.6)
26th to 50th percentile	14,145.0 (24.5)	235.0 (29.0)	14,150 (24.5)	230 (23.1)
51st to 75th percentile	14,710.0 (25.4)	245.0 (30.2)	14,685 (25.5)	270 (27.1)
76th to 100th percentile	12,305.0 (21.3)	185.0 (22.8) *p* = 0.02	12250 (21.3)	240 (24.1) *p* = 0.64
Expected primary payer (%)				
Medicare	30180.0 (51.3)	325.0 (39.6)	29970 (51.1)	535 (53.0)
Medicaid	8665.0 (14.7)	115.0 (14.0)	8660 (14.8)	120 (11.9)
Private insurance	16,400.0 (27.9)	325.0 (39.6)	16,410 (28.0)	315 (31.2)
Self-pay, no charge, and other	3560.0 (6.1)	55.0 (6.7) *p* = 0.007	3575 (6.1)	40 (4.0) *p* = 0.31
Patient Location: NCHS * Urban-Rural Code (%)				
“Central” counties of metro areas of ≥1 million population	16,620.0 (28.3)	230.0 (28.0)	16,585 (28.4)	265 (26.2)
“Fringe” counties of metro areas of ≥1 million population	14,930.0 (25.5)	270.0 (32.9)	14,915 (25.5)	285 (28.2)
Counties in metro areas of 250,000–999,999 population	12,320.0 (21.0)	110.0 (13.4)	12,255 (21.0)	175 (17.3)
Counties in metro areas of 50,000–249,999 population	5470.0 (9.3)	110.0 (13.4)	5490 (9.4)	90 (8.9)
Micropolitan counties	5395.0 (9.2)	60.0 (7.3)	5320 (9.1)	135 (13.4)
Not metropolitan or micropolitan counties	3890.0 (6.6)	40.0 (4.9) *p* = 0.07	3870 (6.6)	60 (5.9) *p* = 0.34
Indicator of a transfer out of the hospital				
Transferred out	11,110.0 (18.9)	75.0 (9.1) *p* < 0.001	11,060 (18.8)	125 (12.4) *p* = 0.02
Weighted Elixir score mean (SD)	16.22 (9.80)	18.02 (8.66) *p* = 0.009	16.23 (9.80)	16.95 (8.96) *p* = 0.26
Length of Stay (Geometric mean)	3.81 days	5.66 days *p* < 0.001	3.81 days	4.95 days *p* = 0.001
Total charge (Geometric mean) *	$56,267	$48,645, *p* = 0.05	$56,267	$42,938, *p* < 0.001

* Abbreviations: SD, Standard deviation; NCHS, National Center for Health Statistics; $, United States’ Dollar; RT, Radiation therapy; Chemo, Chemotherapy; Note: All frequencies and percentages are weighed.

## Data Availability

We used data from the United States’ 2019 National Inpatient Sample database obtained from Healthcare Cost and Utilization Project (HCUP) of the Agency for Healthcare Research and Quality (AHRQ).

## Abbreviations

The following abbreviations are used in this manuscript:

BOI	Burden of illness
CI	Confidence interval
CLOP	Cancers of the lip, oral cavity, and pharynx
Coeff	Coefficient
CT	Chemotherapy
CT-UM	Chemotherapy-induced oral ulcerative mucositis
LOS	Length of stay
RT	Radiotherapy
RT-UM	Radiotherapy-induced oral ulcerative mucositis
UM	Ulcerative mucositis
